# 

**Published:** 1897-07-17

**Authors:** 


### EARLY EDITION.

**Notices of Births, Deaths, and Marriages** are inserted in this column at a charge of 2s. 6d. for an announcement not exceeding four lines.

### NOTICE TO CORRESPONDENTS.

All MS., Letters, Books for Review, and other matters intended for the Editor should be addressed The Editor, "The Hospital," The Hospital Building, 28 & 29, Southampton Street, Strand, W.C.

The Editor cannot undertake to return rejected MS., even when accompanied by stamped directed envelope.

**Notice to Advertisers and Subscribers.**

All Advertisements, Orders for Copies of The Hospital, and Business Communications should be addressed to **THE MANAGER, (Not to The Editor),** The Hospital, The Hospital Building, 28 & 29, Southampton Street, Strand, London, W.C.

The Cost of Subscription, post free, is as follows:—Per week, 2½d.; per quarter, 2s. 8d.; per half-year, 5s. 3d.; per annum, 10s. 6d. Foreign:—Per annum, 15s. 2d.; payable, in all cases, in advance.

**NOTICE.—Vol. XXI. of "The Hospital" (October, 1896, to March, 1897), in handsome cloth binding, is on sale at the Office of this Journal, price 7s. 6d. Cases for binding the Numbers contained in this Volume, 1s. 6d. Index to Vol. XXI., 2½d., post free.**

**The Monthly Part for July is now ready. Price 6d., by post 8d.**

### BOOKS RECEIVED.

William Hodges and Co., Glasgow.

"Annual Report to County Councils of Dumbarton and Stirling." By John C. McVail, M.D., Medical Officer of Health.

John Hogg.

"The Spas of Wales." By T. R. Roberts. Price 1s.

John Wright and Co., Bristol.

"The Pocket Therapist." By Thos. Stretch Dowse, M.D.

E. and S. Livingstone.

"The Insane Poor in Private Dwellings and Licensed Houses." By J. F. Sutherland, M.D., F.R.C.S.E.

Harrison and Son.

"Eighth Annual Report of the Asylum Committee, County Council of Middlesex."

**Periodicals and Pamphlets Received.**—Girl's Own Paper, New York Medical Record, Medical Press, Cassell's Family Magazine, New Age, Literary Digest, One and All, The Zenana, Treatment, The Woman's Signal, Leeds Hospital Magazine, The Councillor, The Vegetarian, The Newsagents and Booksellers' Review, Journal of the Society of Arts, Knowledge, Nursing Notes, Medical Record, London, Guy's Hospital Gazette, London Hospital, Windsor Magazine, The Sun, Local Government Journal, Westminster Review.

### SCRAPS AND GLEANINGS.

The managers of Gray's Hospital, Elgin, have wisely determined to receive no more cases of infectious disease.

Including those in the wards on April 1st, 1896, 1,571 in-patients were treated at the Meath Hospital, Dublin, during the year 1896-7, the mortality being 6·08 per cent. on the total treated to a termination. There were also 4,859 accident cases treated as out-patients, and 11,226 dispensary patients.

### The Student of Medicine.

**APPOINTMENTS.**

A. S. Adams, M.B., C.M.Aberd., appointed Medical Officer for the Rillington District of the Norton Out-relief Union. A. Bethell, M.R.C.S.Eng., appointed Medical Officer of Health to the Bridgnorth Rural District Council. A. G. Butler, M.B.Lond., appointed a House Physician to Guy's Hospital. E. Buxton, M.D., M.R.C.P.E., F.R.C.S.E., D.P.H., appointed Physician to the Nazareth Orphanage at Great Crosby. Mr. W. J. Byford, appointed Assistant Medical Officer to the Nottingham Parish Workhouse. T. G. Crump, B.A.Camb., M.B., B.C., appointed Honorary Surgeon to the Victoria Hospital, Burnley. A. Dingwall, M.B., C.M.Aberd., appointed Medical Officer for the Pembridge District of the Kington Union, and Medical Officer of Health to the Kington Urban District Council. H. P. Ferraby, M.B.Lond., appointed an Assistant House Physician to Guy's Hospital. H. B. Gardner, M.R.C.S.Eng., L.R.C.P.Lond., appointed Assistant Anæsthetist to the Male Lock Hospital, Dean Street, Soho. W. A. Gibb, M.B., M.S., appointed House Surgeon to the Isle of Man General Hospital, Douglas. C. C. Gibbes, M.D., M.R.C.P., D.Ph.Camb., appointed Assistant Physician to the National Hospital for Diseases of the Heart and Paralysis, Soho Square. W. E. Hills, L.R.C.P.Lond., M.R.C.S.Eng., appointed an Assistant House Physician to Guy's Hospital. A. D. Howard, B.A.Camb., M.R.C.S.Eng., L.R.C.P.Lond., appointed Medical Officer for the New Hampton District of the Kingston Union. J. Howell, M.R.C.S.Eng., L.R.C.P.Lond., appointed a House Surgeon to Guy's Hospital. O. J. Kauffmann, M.D.Lond., M.R.C.P., appointed Honorary Physician to the Queen's Hospital, Birmingham. W. Loynd, M.R.C.S.Eng., L.R.C.P.I., appointed Medical Officer for the Oswaldtwistle District of the Blackburn Union. A. J. Martin, M.D.Lond., M.R.C.S.Eng., L.R.C.P.Lond., appointed Honorary Physician to the Walsall and District Hospital. C. A. Morton, F.R.C.S., appointed Joint Professor of Surgery in the Faculty of Medicine of University College, Bristol. Dr. O'Callaghan, appointed Medical Officer for the Ahascragh Dispensary District. R. J. Orford, M.R.C.S., L.R.C.P., appointed Medical Officer of Health to the Market Bosworth District Council. A. W. Ormond, M.R.C.S.Eng., L.R.C.P.Lond., appointed an Assistant House Surgeon to Guy's Hospital. Mr. D. Pratt, appointed Medical Officer for the Odiham District of the Hartley Wintney Union. C. E. Purslow, M.D.Lond., M.R.C.P.Lond., appointed Ingleby Examiner Mason College, Birmingham, for the current year. R. P. Rowlands, L.R.C.P.Lond., M.R.C.S.Eng., appointed a House Surgeon to Guy's Hospital. A. Shillitoe, M.B., B.C.Camb., F.R.C.S.Eng., appointed Surgeon to Out patients at the London Lock Hospital. Mr. R. B. Stamford, appointed an Assistant House Surgeon to Guy's Hospital. A. J. Stiles, M.D.Edin., M.B., C.M., appointed Medical Officer for the Spalding West District of the Spalding Union. R. M. Stokes, L.R.C.P.&S.Edin., L.F.P.&S.Glasg., appointed Medical Officer to the Monmouth District, Monmouth Union. J. Swain, M.S., M.D.Lond., appointed Joint Professor of Surgery in University College, Bristol. Mr. F. G. Thomas, appointed a House Physician to Guy's Hospital. W. M. Woodhouse, M.R.C.S.Eng., L.R.C.P.Lond., appointed Medical Officer for the Vale District of the Wantage Union.

